# Gold as a Material for Filling Teeth

**Published:** 1891-12

**Authors:** R. E. Sparks

**Affiliations:** KINGSTON, ONT.


ARTICLE IV.

### GOLD AS A MATERIAL FOR FILLING TEETH.

R. E. SPARKS, L. D. S., KINGSTON, ONT.

Much has been, and is being written upon the subject of materials best adapted for the successful filling of teeth. Owing to their malleability, lead and tin were used with considerable success before gold in its present forms and the plastic fillings, now so popular, were prepared.

Gold was the next material which came into general use, and is said to have been used at a very early date. But only during the present century has the operation of preserving the natural teeth, by means of filling the cavities made by decay, been very generally performed with any marked degree of success.

So wedded to gold, as a filling material, were the old operators of the present generation, that it became an axiom with them, that a tooth which was worth filling at all was worth filling with gold. The man who dared to suggest that plastic material might be used to advantage in certain cases was set down as a crank or charlatan. Gold possessed advantages over the other then known materials. Lead and tin were soft, and consequently became worn away by the friction of mastication. Their dull color showing through thin enamel walls gave the teeth a discolored appearance. Gold was dense and withstood friction well. It also retained its color and brilliancy. So late as the edition of 1871 of "Harris' Principles and Practice of Dentistry," the author says of gold: "It is the only material in the opinion of the author which should be employed for the permanent filling of teeth."

Gold was first used as non-cohesive foil, and, to be retained in the cavity, required retaining pits or grooves in almost all directions. The contour fillings, which are the pride of so many operators, were wholly unknown. It was not until Dr. Robert Arthur wrote a treatise on "The use of adhesive foil," as late as 1857, and demonstrated the applicability of this form of gold in filling teeth, that the use of cohesive foil became at all general. If gold was found to be such a valuable filling material when used as non-cohesive only, what may be said of its value in its cohesive form, when a cavity having two walls, or even one wall, well grooved, will retain a filling? or when a tooth having lost a part, or even the whole crown, may be restored to its proper contour and articulation?

But while some men have gone wild over gold as a filling material, and held the opinion that gold only should be used, others have gone to the other extreme, and say gold should never be used. Some who once lauded gold up to the sky, now condemn it. A prominent dentist of Philadelphia, who was once a great advocate of gold, now, it is said, has printed upon his cards, "No gold used." While it is wise to profit by the experiences of others, what are we to do with such conflicting evidence? We must then draw upon the evidence of our own experience. Let us look at the requirements of a variety of cavities. I once heard Dr. Jas. H. Harris, of Baltimore, clinical professor of the dental department of the University of Maryland, say, of the relative merits of cohesive and non-cohesive gold foil, "It is impossible for an operator to do justice to himself or his patients by confining himself to the exclusive use of either form." I would say the same, from my own experience of gold vs. other materials. A case is presented: A central incisor, healthy patient, good tooth, pulp well covered, plenty of room for retention; that case calls for gold. Another case: A central incisor, patient very delicate, of lymphatic temperament, enamel of tooth thin, labial and palatine walls gone, tooth extremely sensitive; that tooth calls for some preparation of gutta percha or zinc. Another case: A cavity on the posterior approximal surface of a molar; if the condition of the tooth would warrant the use of a metal filling at all, that case demands amalgam. In that position the discoloration of amalgam would not be an objection, and a more perfect filling could be made than could be made of gold in the same position, and with much less fatigue to both patient and operator.

To sum up. The advantages of gold are: density, enabling it to withstand the friction of mastication well. Indestructibility, enabling it to withstand the action of ordinary solvents. Cohesiveness, enabling it to restore the contour and articulation of parts of teeth which may have been destroyed. Adaptability, permitting it to be so closely packed to the walls of a cavity that moisture may be excluded. Retention of color, neither becoming discolored nor discoloring the tooth.

Its disadvantages are: its color, making it a very conspicuous filling. Its conductivity, making it an unsafe material with which to fill a cavity, having an exposed pulp, which is very sensitive. Its inadaptability, making it (1) Tedious to insert, for, to adopt it perfectly to the walls of a cavity, it must be inserted bit by bit. (2) Expensive, because, for his time, rather than for the material used, does the operator expect remuneration. (3) Leaky, because cavities often present themselves which it is next to impossible to fill perfectly with gold, owing to their inaccessibility, or to the difficulty with which they may be kept dry. Who has not seen large compound approximal surface cavities in bicuspids or molars, containing apparently fine gold fillings, but which weie leaky at the cervical wall? Such a filling is patched with gold with much more difficulty than the original cavity was filled, and with much more uncertainty of success. Therefore, I would recommend gold for front teeth of good quality, and for back teeth, where the cavities are small and easily reached; for, in such cavities, I consider the advantages to counterbalance the disadvantages of gold as a filling material.—*Dominion Dent. Journal.*

